# Social Media Data Analytics on Telehealth During the COVID-19 Pandemic

**DOI:** 10.7759/cureus.7838

**Published:** 2020-04-26

**Authors:** Elie Massaad, Patrick Cherfan

**Affiliations:** 1 Postgraduate Research, Harvard Medical School, Boston, USA; 2 Faculty of Medicine, American University of Beirut, Beirut, LBN

**Keywords:** natural language processing, data mining, telehealth, covid-19, twitter

## Abstract

Introduction: Physical distancing during the coronavirus Covid-19 pandemic has brought telehealth to the forefront to keep up with patient care amidst an international crisis that is exhausting healthcare resources. Understanding and managing health-related concerns resulting from physical distancing measures are of utmost importance.

Objectives: To describe and analyze the volume, content, and geospatial distribution of tweets associated with telehealth during the Covid-19 pandemic.

Methods: We inquired Twitter public data to access tweets related to telehealth from March 30, 2020 to April 6, 2020. We analyzed tweets using natural language processing (NLP) and unsupervised learning methods. Clustering analysis was performed to classify tweets. Geographic tweet distribution was correlated with Covid-19 confirmed cases in the United States. All analyses were carried on the Google Cloud computing service “Google Colab” using Python libraries (Python Software Foundation).

Results: A total of 41,329 tweets containing the term “telehealth” were retrieved. The most common terms appearing alongside ‘telehealth’ were “covid”, “health”, “care”, “services”, “patients”, and “pandemic”. Mental health was the most common health-related topic that appeared in our search reflecting a high need for mental healthcare during the pandemic. Similarly, Medicare was the most common appearing health plan mirroring the accelerated access to telehealth and change in coverage policies. The geographic distribution of tweets related to telehealth and having a specific location within the United States (n=19,367) was significantly associated with the number of confirmed Covid-19 cases reported in each state (p<0.001).

Conclusion: Social media activity is an accurate reflection of disease burden during the Covid-19 pandemic. Widespread adoption of telehealth-favoring policies is necessary and mostly needed to address mental health problems that may arise in areas of high infection and death rates.

## Introduction

The novel coronavirus outbreak that started in Wuhan, China entered a new phase after the World Health Organization (WHO) officially declared it as a global pandemic on March 11, 2020 [1]. This called out for urgent changes in the medical sector after presuming hospital-associated transmission in nearly 30% of infected health professionals and 12% of hospitalized patients [2]. Telehealth rapidly became a necessary technology to guarantee continuity of care amidst worldwide physical distancing policies, by allowing patients to receive medical care while minimizing the risk of exposure - a critical concern for the elderly and those with chronic conditions [3]. As the pandemic remains ongoing, urgent action is required to support the digital transformation in healthcare and to understand the various concerns of patients needing care during this crisis [4]. Healthcare workers, patients, institutions, technology industries, and policymakers turned to social media to embrace this rapid shift in healthcare delivery. Twitter (Twitter, Inc., San Francisco, CA) has been considered among the best social media platforms for keeping its users on top of the most trending topics and in understanding consumers’ opinions on health technology matters [5]. For this reason, we planned to explore the data available on social media to better characterize the surge in telehealth during the Covid-19 pandemic. Our study aims to analyze the dynamics of social media data related to telehealth and understand the public activity to strategically optimize and accelerate the digital health transformation.

## Materials and methods

Study design and data collection

This cross-sectional study was conducted from March 30, 2020 to April 6, 2020. This study was exempted from the Institutional Review Board at Harvard Medical School because the data used are publicly available. In this instance, a consent form was not necessary. This study followed the Strengthening the Reporting of Observational Studies in Epidemiology (STROBE) reporting guidelines.

Twitter is an online public social media platform that allows users to post 280-character posts. Publicly posted “tweets” from March 30, 2020 to April 6, 2020, were collected through the public streaming API (application programming interface).

Text mining

Full-text tweets were preprocessed by converting the sentences to words (Tokenization), removing unnecessary punctuations, tags, and stop words that do not have a specific semantic meaning (i.e. “the”, “are”). We applied a stemming function with lexicon normalization to reduce related words to a common word root (i.e. connection, connected, connecting were reduced to “connect”). We isolated tweets with an identifiable location within the United States and constructed a density map. Preprocessing was done using the Natural Language Toolkit (NLTK) on Python 3.0 [6]. 

Mapping Covid-19 confirmed cases

Information on Covid-19 cases in the United States was obtained from the freely available public database (https://github.com/CSSEGISandData/COVID-19) published by the Center for Systems Science and Engineering (CSSE) at Johns Hopkins University, Baltimore, MD, USA. The numbers presented in this manuscript were posted on April 4, 2020. The U.S. map illustrating the number of confirmed Covid-19 cases in each state was generated using Plotly; a Python open-source graphing library [7].

Statistical methods

Descriptive analytics were performed to study the data collected. Tweet characteristics included account median number and range of followers. Generalized linear regression was performed to study the association between “telehealth” tweets and the number of confirmed Covid-19 infections. Statistical significance was set at p<0.05. Unsupervised learning was performed using K-means clustering algorithms to classify tweets into topics. We used the Elbow method to define the number of K dimensions where K depends on the number of topics. All data preprocessing, analysis, and visualization were performed on the Google Cloud computing service “Google Colab” (colab.research.google.com) using Python 3.0 programming language (Python Software Foundation; http://www.python.org).

## Results

Social media data characteristics

Our search revealed that the word “telehealth” appeared in 41,329 posts on twitter from March 30, 2020 to April 6, 2020. The retrieved Twitter accounts had a median number of followers of 726 (range 0-12 509 982). Every post containing the word “telehealth” was retweeted with a median number of two times (range 0-7556). The 10 most common words apart from “telehealth” that appeared in these tweets were “COVID”, “health”, “care”, “services”, “patients”, “pandemic”, “coronavirus”, “healthcare”, “access”, “need”. Mental health was the most common health-related topic that appeared in our search. Similarly, Medicare was the most common appearing health policy-related topic mirroring the accelerated response to telehealth and the changes in coverage policies. Unsupervised machine learning classification of tweets identified six clusters of tweets that contained words mostly related to: (1) mental health services, (2) digital health, (3) policies and advocacies, (4) hydroxychloroquine, (5) technology, and (6) general opinions.

Geospatial distribution of tweets in the United States

**Figure 1:**
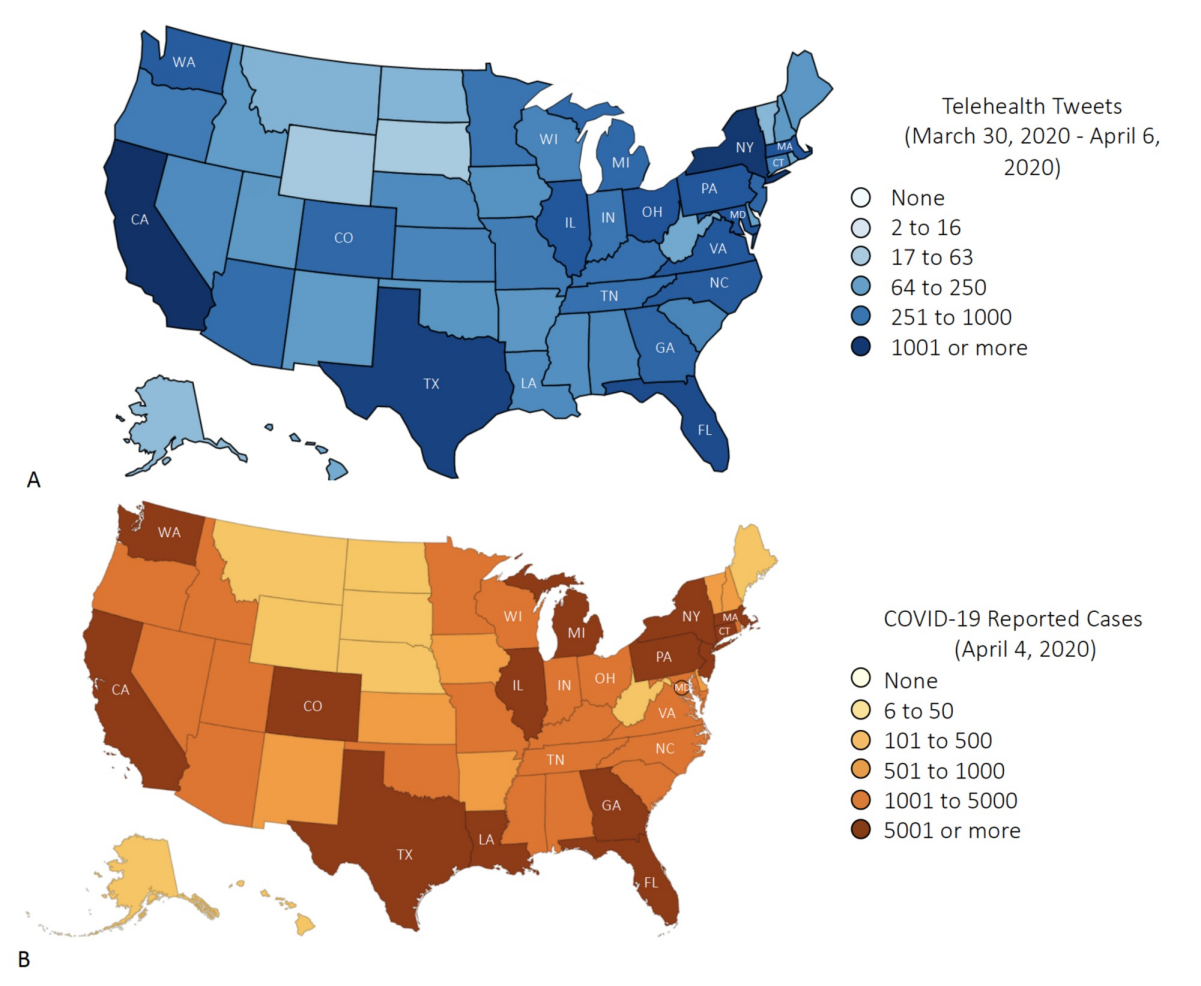
. A. State-specific number of tweets containing the word “telehealth”. B. State-specific prevalence of coronavirus COVID-19 cases. The 20 states with the highest numbers of infection are represented by their postal code on both maps.

In the United States, our analysis showed that tweets were most commonly posted from California (CA), New York (NY), Texas (TX), Florida (FL), and Pennsylvania (PA). In contrast, among the 20 most common tweeting U.S locations, tweets having “telehealth” were less likely to be posted from Tennessee (TN), Indiana (IN), Michigan (MI), Minnesota (MN), and Maryland (MD). The geographical distribution of tweets was illustrated on a U.S. density map (Figure 1A).

In parallel, New York (NY) State has recorded the highest number of confirmed cases, followed by New Jersey (NJ), Michigan (MI), and California (CA). Among the 20 most infected states in the United States, Wisconsin (WI), North Carolina (NC), Maryland (MD), Tennessee (TN), Ohio (OH), and Indiana (IN) have the lowest numbers of confirmed cases (Figure 1B).

**Figure 2:**
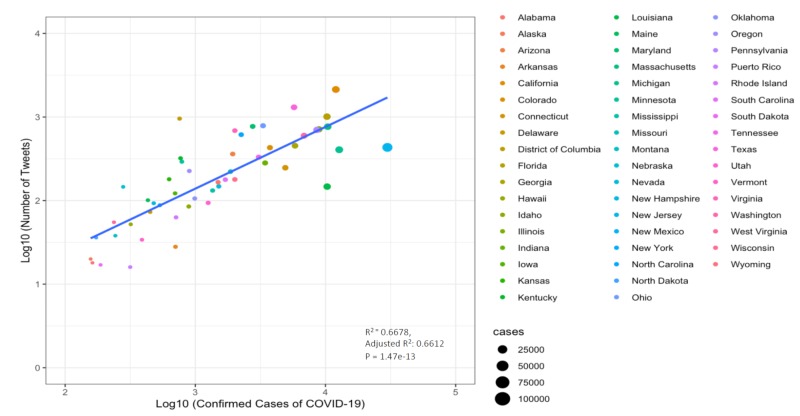
Scatter plot of the log10 (number of tweets) on log10 (number of confirmed coronavirus COVID-19 cases).

Our analysis revealed an association between the number of tweets related to telehealth posted in a certain state and the number of confirmed Covid-19 cases in that particular state (p<0.001) (Figure 2). This variable distribution can also be visualized by comparing the density map with the number of tweets to that with the number of confirmed Covid-19 cases in each state.

## Discussion

Social media platforms have proven to be very engaging with the public [8]. Data analysis of social media content has provided insight into political campaigns, media, healthcare, and daily events [9]. In this study, we retrieved and analyzed public data available on Twitter to investigate the rapid shift in telehealth adoption amidst the recent coronavirus Covid-19 pandemics. Our results highlighted the need for widespread implementation of digital health and the importance of favoring policy changes to unleash the power of this technology. Interestingly, the number of tweets related to telehealth was associated with the number of Covid-19 cases in each of the U.S. states. Moreover, mental health appeared to be the most common health-related issue discussed online. This may refer to the significant effects on mental health in areas of high disease burden [10].

In fact, efforts to implement telehealth were rapidly mobilized across the United States on many levels. Healthcare plans and agencies provided regulatory relief and reimbursement policies to provide telehealth services during this public health emergency [11]. Major Medicare and Medicaid telehealth policy updates in the 10 states with the highest reported coronavirus positive cases are summarized (Table 1). Physical distancing and shelter-in-place orders are likely to result in considerable psychological distress, which prompts healthcare providers and organizations to ensure and sustain a pandemic workforce that addresses crisis-related health problems [12].

**Table 1:** Summary from the Center for Connected Health Policy on the 10 states with the highest reported coronavirus positive cases.

State	Current telehealth laws and reimbursement policies
New York	-Reimbursement for live video -Some reimbursement for store-and-forward and home health services
New Jersey	-Reimbursement for live video and remote patient monitoring under certain circumstances
Michigan	-Reimbursement for live video telemedicine for certain healthcare professionals, for patients located at certain originating sites -No reimbursement for store-and-forward or remote patient monitoring
California	-Providers allowed to decide what modality, live video or store-and-forward, will be used to Medical enrollees
Massachusetts	-No reference to reimbursement of telehealth or telemedicine within Mass Health policies
Louisiana	-Live video telemedicine covered for distant site providers -No reimbursement for the originating site
Florida	-Reimbursement for real time interactive telemedicine according to administrative code -No reference for store-and-forward or remote patient monitoring.
Illinois	-Reimbursement for live video telemedicine and telepsychiatry services for specific providers
Pennsylvania	-Reimbursement for live-video under some circumstances -No reimbursement for store-and-forward or remote patient monitoring
Washington	-Reimbursement for live video, store-and-forward, and remote patient monitoring under some circumstances

## Conclusions

Telehealth services have rapidly and largely transformed healthcare delivery in areas of high infection rates. In parallel, social media platforms became an immense source of information on day-to-day events and a reflection of social interactions and responses. In this study, we showed that such platforms can be used to assess the needs of our communities and to embrace the healthcare response, resilience, and preparedness during pandemics. Nationwide efforts should focus on lifting provisions and scaling up resources to expand digital health implementation to address crisis-related sequelae. 
